# Supplementary material for: Rigid head-neck responses to unpredictable perturbations in patients with long standing neck pain does not change with treatment
Source: PLoS One. 2020 Aug 24;15(8):e0237860. doi: 10.1371/journal.pone.0237860 (PMC7446813; doi:10.1371/journal.pone.0237860)
Supplement: S1 File — (DOCX) [file pone.0237860.s001.docx]

**Appendix 1**

The sum-of-sines angular velocity excitation signal $u\left( t \right)$ is defined as

$u\left( t \right)=\sum_{k\in H} a_{k}\sin\left( 2\pi Fkt+\phi_{k} \right)$,

where $k$ represents each of the harmonics in the set $H= \left\{ 37, 49, 71, 101, 143, 211, 295, 419, 589, 823 \right\}$, $a_{k}$ is the amplitude of the $k$’th harmonic in radians/second, $t$ is time in seconds, and $\phi_{k}$ is the phase angle in radians of the $k$’th harmonic at $t=0$.

In our case $\phi_{k}=0\forall k$, but actual values used are considered irrelevant for the results, thus $\phi_{k}$ is left out of the equations in the following.

Excitation induced sinusoidal rotations in the trunk and head are given by the formulae

$$\theta^{T}\left( t \right)\approx\sum_{k\in H} A_{k}^{T}\sin\left( 2\pi Fkt \right)$$

$$\theta^{H}\left( t \right)\approx\sum_{k\in H} A_{k}^{H}\sin\left( 2\pi Fkt \right)$$

where ($T$) is the trunk and ($H$) is the head angle, and $\theta^{T}$ and $\theta^{H}$ are the trunk-room angle and the head-room angle, respectively. Approximation signs are used to account for noise and possible harmonic distortions.

The excitation signal is defined as the angular velocity $u\left( t \right)$, while $\theta^{T}(t)$ and $\theta^{H}(t)$ are rotation angles. The angular excursion amplitude coefficients $[A_{k}]$ relate to the angular velocity amplitude coefficients as $A_{k}=-\frac{1}{2\pi Fk}a_{k}, k\in H$.

**Appendix 2**

The transfer function of a system can be defined as the ratio between the system’s response and the corresponding excitation. In particular, a linear dynamic system’s transfer function can be represented as a complex function $G$ such that

$G\left( j2\pi f \right)=\frac{Y(j2\pi f)}{U(j2\pi f)}$,

where $Y$ and $U$ complex functions representing the response and the excitation, respectively, $j$ is the imaginary unit and $f$ is frequency in Hz.

In the following analysis we regard the measured trunk-room angle $\Theta^{T}(t)$ as the excitation and the resulting measured head-room angle $\Theta^{H}$ as the response. The following integrals furnished complex signal descriptions of the $k$’th harmonic of each of these signals:

$$\Theta_{k}^{T}=\frac{2}{T}\int_{0}^{T} \theta^{T}\left( t \right)e^{-j2\pi Fkt}dt, \Theta_{k}^{H}=\frac{2}{T}\int_{0}^{T} \theta^{H}(t)e^{-j2\pi Fkt}dt, k\in H$$

The transfer function was subsequently evaluated at the discrete excitation frequencies by evaluating

$$G_{k}=\Theta_{k}^{H}/\Theta_{k}^{T}, k\in H$$

Gain and phase shifts of the head-room angle relative to the trunk-room angle were recovered by taking the absolute value and argument (angle) of this complex transfer function, as follows:

$$\frac{A_{k}^{H}}{A_{k}^{T}}=\left| G_{k} \right|, \phi_{k}^{H}-\phi_{k}^{T}=\arg G_{k}$$

Resulting transfer functions are presented as Bode plots with gain and phase shown for the 10 excitation frequencies. The Bode plot (with decimal logarithmic gain and linear phase) decouples the system properties of gain, phase shift and time constants/Eigen-frequencies, which allow direct comparison and statistical analyses of linear systems with different dynamics, expected in human bodies of different size and mass. Thus, our statistical analysis is also based on Bode data.
